# Provision of acute medicine services for pregnant women in UK hospitals: Data from the Society for Acute Medicine Benchmarking Audit 2019

**DOI:** 10.1177/1753495X20929502

**Published:** 2020-06-23

**Authors:** Catherine Atkin, Paarul Prinja, Anita Banerjee, Mark Holland, Dan Lasserson

**Affiliations:** 1 Institute of Inflammation and Ageing, University of Birmingham, Birmingham, UK; 2 Royal Wolverhampton NHS Trust, Wolverhampton, UK; 3 Guy’s and St Thomas’ NHS Foundation Trust, London, UK; 4 Salford Royal NHS Foundation Trust, Salford, UK; 5 Institute of Applied Health Research, University of Birmingham, Birmingham, UK; 6 Department of Acute Medicine, City Hospital, Sandwell and West Birmingham Hospitals NHS Trust, Birmingham, UK

**Keywords:** Pregnancy, postpartum, acute care

## Abstract

**Background:**

Medical problems during pregnancy are the leading cause of maternal mortality in the UK. Pregnant women often present through acute services to the medical team, requiring timely access to appropriate services, physicians trained to manage medical problems in pregnancy, with locally agreed guidance available.

**Methods:**

Data were collected through the Society for Acute Medicine Benchmarking Audit, a national audit of service delivery and patient care in acute medicine over a 24 hour period.

**Results:**

One hundred and thirty hospitals participated: 5.5% had an acute medicine consultant trained in obstetric medicine, and 38% of hospitals had a named lead for maternal medicine. This was not related to hospital size (p = 0.313). Sixty-four units had local guidelines for medical problems in pregnancy; 43% had a local guideline for venous thromboembolism in pregnancy. Centres with a named lead had more guidelines (p = 0.019).

**Conclusion:**

Current provision of services within acute medicine for pregnant women does not meet national recommendations.

## Introduction

Maternal mortality remains an important cause of death for young women in the United Kingdom, with a current rate of 9.8 deaths per 100,000 pregnancies.^1^ Mortality is due to both direct causes which are attributable to the pregnancy, and indirect causes including pre-existing medical problems or those occurring de novo during pregnancy. Venous thromboembolic disease (VTE) is the most common direct cause of mortality, and cardiac disease remains the most common indirect cause in the UK.^2^ Pregnant or recently pregnant women with acute illness attending secondary care frequently present through emergency services to the acute and/or general medical team for initial investigation and management. Acute and general medical on-call services within secondary care will therefore be the most commonly used care pathway for management of acute deterioration.

The Mothers and Babies: Reducing Risk through Audit and Confidential Enquiries (MBRRACE-UK) reports provide annual review of maternal deaths within the UK.^2^ This provides a growing body of recommendations on how medical care should be delivered during and after pregnancy. This mortality reflects the poorest outcomes from a larger burden of disease requiring specialist management during pregnancy, including pre-existing or de novo medical problems at risk of deterioration during pregnancy, such as cardiac disease, respiratory disease and epilepsy.

Pregnant women should receive the same care as they would if they were not pregnant, except where it would cause harm.^2^ For most patients presenting with an acute medical emergency or deterioration of chronic disease, the most appropriate place for assessment and management by the medical team is the acute medical unit (AMU) or ambulatory emergency care (AEC), following referral from the emergency department or primary care.^3^ These assessment units provide initial stabilisation, investigation and treatment of acute illness, with care provided by the on-call acute or general internal medicine team. AEC aims to provide these services within the same day, discharging patients home safely without inpatient admission.

MBRRACE-UK recommend pregnant women presenting with acute illness be reviewed by appropriately trained senior physicians, with involvement of the hospital’s maternal medicine team. Although obstetric trainees are able to undertake subspecialty training in maternal and fetal medicine, recognised by the Royal College of Obstetricians and Gynaecologists (RCOG),^4^ many obstetricians will not have had specialty training in obstetric medicine. Physician training in acute or general internal medicine includes medical problems in pregnancy within the curriculum, requiring competency in ‘assessment, investigation and management of the common and serious medical complications of pregnancy’,^5^ but many physicians will not have had specific experience of obstetric medicine and obstetric medicine does not currently have a stand-alone physician training pathway through the Royal Colleges in the same manner as other medical specialties. Trainees in Acute Internal Medicine can choose to undertake specialist skill training in obstetric medicine, with specific training and assessment in obstetric medicine during their specialty training.^6^

It is recommended that hospitals have specific guidelines for the management of medical problems in pregnancy. Although RCOG guidelines provide nationally approved guidance, guidelines specific to local pathways are required, including the diagnosis and management of VTE.^7^

Each year the Society for Acute Medicine (SAM) conducts a benchmarking audit to assess the structure and delivery of acute medical care within hospitals in the UK, including a 24 h ‘day of care’ survey for all newly assessed and treated patients.^8^ We utilised this audit to assess the current provision of obstetric medical care within AMUs in the UK.

## Methods

Data were collected through the Society for Acute Medicine Benchmarking Audit (SAMBA) performed in June 2019. The audit includes patients admitted over one 24 h period, collecting data determined by national quality standards for processes of care as well as national guidelines, NHS England recommendations or professional society guidance. This includes time from attendance to initial assessment and consultant review. Data are also collected regarding the size and staffing of each acute medical department, and the size of the hospital.

Participation in SAMBA is voluntary, and departments register through an online portal via SAM. The audit was registered locally at each site. All data are collected locally, anonymised and transferred to the central database held at the Royal College of Physicians of Edinburgh.

The obstetric medicine questions in the 2019 audit were chosen to reflect recommendations from previous MBRRACE-UK reports, particularly focussing on the availability of trained staff, access to acute services and availability of specific guidelines.

The questions included were as follows:

- Does your hospital have maternity services on site?
- Where are women who are pregnant or up to six weeks postpartum seen with acute medical problems? (select all relevant options from AMU, AEC, maternity assessment unit)
- What is the maximum gestation that you see on AMU/AEC?
- How many acute medicine consultants in your unit have undergone special skills training in maternal medicine?
- Is there a named lead for maternal medicine in your trust?
- For acute medical problems in pregnancy, do you have any condition or presentation specific guidelines?

The results were analysed using descriptive statistics. Comparisons were made using non-parametric tests as data were not normally distributed.

## Results

### Unit organisation

In total 141 units, which were part of 130 individual hospitals, participated in SAMBA 2019 with 7170 patients included.

**Figure 1.**
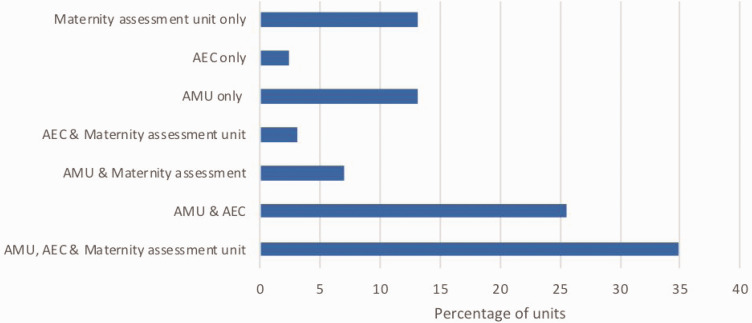
Locations used for assessment of pregnant women by the medical team. AEC: ambulatory emergency care; AMU: acute medicine unit.

One hundred and thirty hospitals answered the questions regarding obstetric medicine, with on-site maternity services at 85.2% of these sites. All 130 units reported that the acute medicine team provided care for pregnant women. The locations used to assess pregnant women are shown in Figure 1. 13.2% of units did not see pregnant women in AMU or AEC, reviewing pregnant women only on the maternity assessment unit within their maternity services.

**Figure 2.**
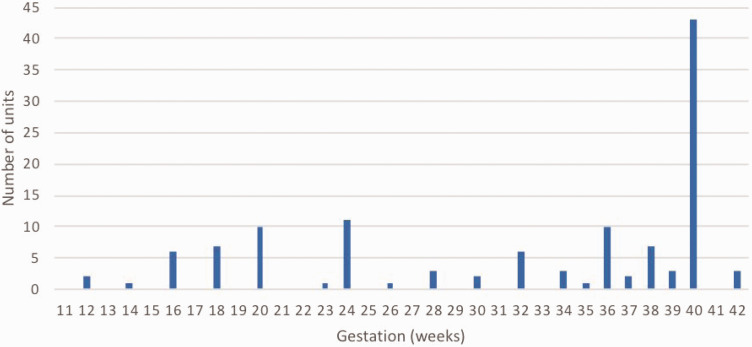
Gestation-based cut-offs for admission to acute medicine department. Gestation by weeks; number of units reporting each cut-off point shown. No unit reported a gestation-based cut-off point below 12 weeks.

Reported cut-off points for review in acute medicine departments at each hospital based on gestation varied (Figure 2); 46% of departments saw pregnant women up to or past term (37+ weeks’ gestation).

Seven units (5.5%) had an acute medicine consultant with specialist skill training in obstetric medicine. No unit had more than one acute medicine consultant with obstetric medicine training.

Forty-nine centres (38.3% of units) had a named lead for maternal medicine in the hospital trust. There was no difference in size of hospital between those units with and without a named maternal medicine lead (Table 1).

**Table 1.** Influence of hospital size on presence of maternal medicine lead and number of guidelines. Comparison for named maternal medicine lead using Mann–Whitney U test; comparison for number of local guidelines using Kruskal–Wallis test.

	Size of hospitalMedian number of beds (interquartile range)		p value
Named maternal medicine lead (n = 129)			
Yes	527	(386–828)	0.313
No	525	(372–698)	
			
Number of local guidelines (n = 123)			
0	465	(377–678)	0.161
1	500	(318–712)	
2+	601	(466–850)	

**Figure 3.**
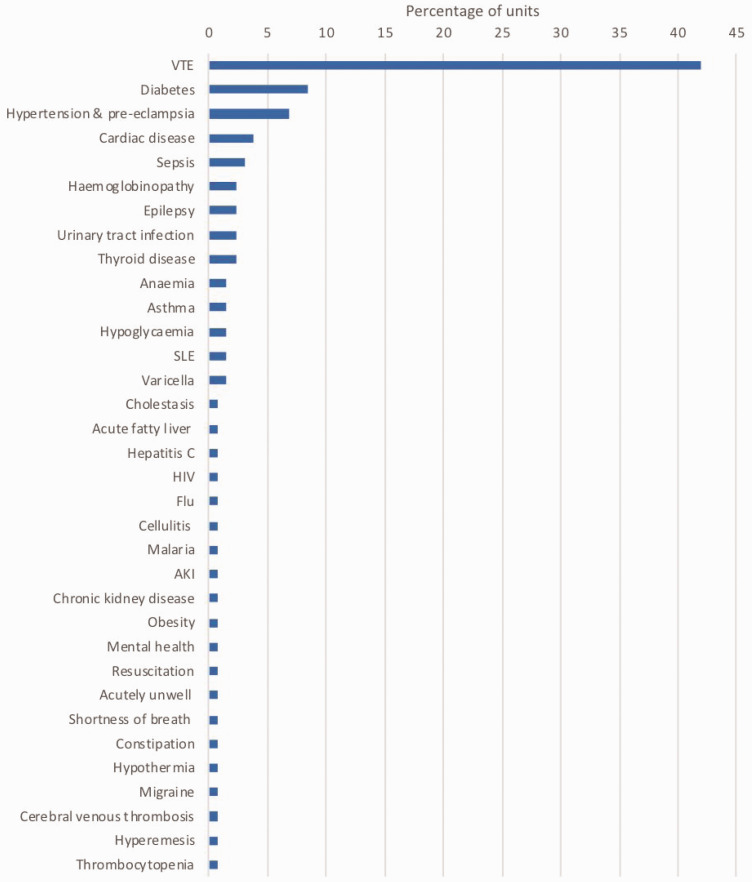
Availability of local guidelines for medical problems in pregnancy. Percentage of units reporting each guideline. AKI: acute kidney injury; HIV: human immunodeficiency virus; SLE: systemic lupus erythematosus; VTE: venous thromboembolism.

Condition specific guidelines for medical problems in pregnancy were available in half of units (64/127, 50.4%). A local guideline specific to VTE in pregnancy was available in 43%. The range of guidelines available is shown in Figure 3. There was no significant difference in the size of hospital comparing units with no guidelines, one guideline or more than one guideline (Table 1). Centres with a named maternal medicine lead had a higher median number of guidelines than those without (median 1 versus 0, Mann–Whitney U test, p = 0.019).

### Women seen on day of the audit

Fifty-three women seen on the day of data collection were pregnant. This was 5.3% of women aged 16–49 years seen that day.

**Figure 4.**
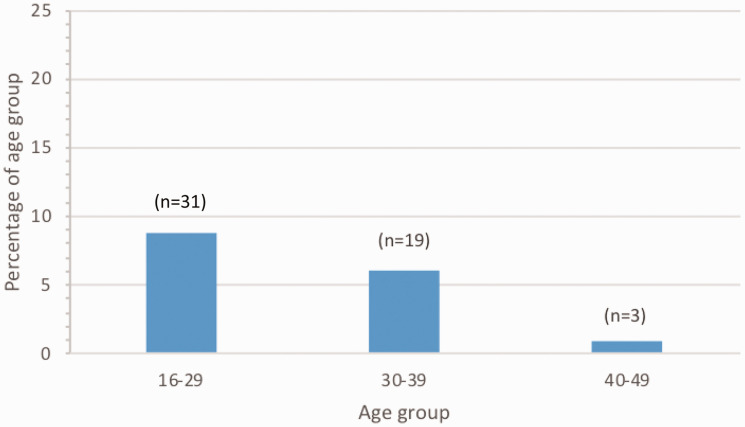
Percentage of women of childbearing age who were pregnant. Number of pregnant women in each age group also shown.

58.5% of the pregnant women seen were 16–29 years old. The percentage of each age group of women who were pregnant is shown in Figure 4.

Planned re-attendance to AEC accounted for 18.9% of pregnant women seen (95% CI 10.6–31.4%). This was not significantly different to the proportion of non-pregnant women of the same age who were planned reviews (11.8%, 95% CI 9.9–14%). 26.4% (95% CI 16.4–39.6%) had been seen at a hospital in the preceding 30 days, which was not significantly different to the percentage of non-pregnant women (17.7%, 95% CI 15.6–20.4%).

Assessing arrival time, 94.3% arrived in daytime hours between 08:00 and 20:00 (95% CI 84.6–98.1%). This was a higher proportion than non-pregnant women of the same age (79.9%, 95% CI 77.2–82.3%).

Initial review was within the Emergency Department for 32.1% of pregnant women, with 15.1% assessed directly in the AMU, and 52.8% assessed in AEC.

## Discussion

All acute medicine departments that participated in SAMBA provided care for medical problems during pregnancy; however, there was variation in the services available in each centre. Only seven centres (5.5%) had an acute medicine consultant with specialist training in obstetric medicine, and a named lead for maternal medicine was available in 49 centres (38.3%). Only half of centres had guidelines specific to the management of medical problems in pregnancy.

These results suggest several areas where the provision of care for pregnant women presenting with acute medical problems falls behind current recommendations.

All services provided acute medical care for pregnant women, and 1 in 20 women of childbearing age was pregnant, with higher rates in younger women. This therefore represents a group that will be frequently encountered by the general medical team, and so access to appropriate services and trained staff is needed within these departments.

Pregnant women were more likely to present during the daytime, with a large percentage presenting through AEC services. However, there was variation in where patients were seen, including whether they could be admitted to AMUs or access AEC. The high percentage of pregnant women seen in AEC, including attendance for planned reviews, suggests there is demand for these services within this group and trained staff and appropriate guidelines must be available. During pregnancy, women should be able to access the same medical care as all other patients, which may include the use of AEC to access outpatient investigation and treatment pathways.^9^

As well as variability in assessment location, there was also variability in the maximum gestation admitted to the acute medical department. This variability suggests that a pregnant woman may have access to different medical care based on which hospital she attends. Setting gestation-based limits when deciding where to admit women may also lead to difficulty when women are found to be pregnant only after admission, for example on pregnancy testing before procedures or imaging.^10,11^ How these admission criteria were determined could not be assessed during this project, for example whether they reflect local capabilities to deal with deliveries of varying gestational age, and these factors need to be explored further.

Only 5.5% of centres had an acute medical consultant that had undergone specialist skill training in obstetric medicine, and no centre had more than one acute medicine consultant with this training. Without trained consultants to supervise trainees undertaking this specialist skill, it is more challenging for trainees to complete, and as this training is self-organised, this may discourage trainees from choosing this training.^6^

Only half of centres reported having a named lead for maternal medicine. There was no difference in hospital size between those with a named lead and those without, therefore this is not a problem affecting only small hospitals with less specialised services available locally. Data were not collected on the specialty of the named lead, and many of these may be obstetricians with training in maternal medicine or may be physicians trained in medical specialties other than acute medicine. This figure may be an underestimate if data collectors were not aware of the lead for maternal medicine in their trust. However, data for SAMBA are collected by the acute medical team themselves, and if they are not aware of their local referral routes and pathways then they are less likely to use them.^12^

Half of centres had specific guidelines for the management of medical problems in pregnancy. Focussing specifically on VTE, only 43% had local guidance. While there are national guidelines available on the management of VTE in pregnancy,^7^ the MBRRACE reports are clear in the recommendation of local guidelines.^2^ Local guidelines and pathways are important as they can be adapted to reflect the services available at each site, such as access to imaging and referral pathways to specialist centres where specific skills are not available in smaller hospitals.^13^ Although there were a wide range of other guidelines for specific problems in pregnancy, these were often seen in only one centre, suggesting localised pockets of knowledge that may not be shared. Centres with a named maternal medicine lead had a higher number of guidelines available, suggesting these centres may have a more developed service offered locally.

Overall, there is considerable variability in the care that women may receive depending on location within the UK. This variability needs to be explored further to assess the impact on those presenting with medical problems in pregnancy. As SAMBA is a day of care audit that takes place over one 24 h period, it may not fully capture how acute care services are utilised by pregnant women.

Severity of illness in SAMBA was assessed by the National Early Warning Score on admission. This scoring system is not validated for use in pregnancy due to altered physiology,^14^ where the Modified Early Obstetric Warning Score (MEOWS) can be used.^15^ As there is currently no standardised national MEOWS in use, it was not included in SAMBA. The reason for admission was not recorded, therefore the range of presentations or conditions seen could not be assessed.

Although these are valuable data about current provision of obstetric medicine in acute medical care, this may not fully capture the pathways used or underlying reasons why pathways have developed in this way.

Since these data were collected, the Royal College of Physicians has published an acute care toolkit (Table 2).^16^ This provides recommendations where provision could be assessed through SAMBA in future. Included in these recommendations is that each AMU has a named clinician to act as liaison with the obstetric team. In view of the low number of acute medicine consultants with specialist skill training in obstetric medicine, these named liaisons are unlikely to have specialist skill training. It will likely take time for the number of acute medicine consultants with specialist training in obstetric medicine to grow, due to the time to train new consultants and the limited number of trained acute medical consultants available to supervise. Establishing national links between those acute medical consultants working in obstetric medicine and sharing knowledge and skills may help to encourage trainees to consider obstetric medicine and to improve local services including the provision of pathways and guidelines.^17^

**Table 2.** Key recommendations from Royal College of Physicians Acute Care Toolkit 15: Managing acute medical problems in pregnancy.

Key recommendations from Royal College of Physicians & Society for Acute Medicine
• Named clinical lead from acute medicine to liaise with obstetrics • Named clinical lead from obstetrics to liaise with acute medicine• Contact details for emergency obstetrics on-call or midwife readily available to staff on the acute medical unit (AMU) • All clinical staff receive ongoing education and training in the management of acute medical problems in pregnancy and the postpartum period (including use of MEOWS) • Escalation measures in place for the acute deterioration of a pregnant woman• Local inpatient shared care pathways/services in place for pregnant women presenting with acute medical problems, including where they should be cared for• Local clinical guidelines available for staff looking after pregnant women presenting with acute medical problems• Joint inpatient medical and obstetric care for women with complex medical problems (such as inflammatory bowel disease, connective tissue diseases, cardiac disease) and acute medical problems where a decision may need to be taken regarding timing of delivery

MEOWS: Modified Early Obstetric Warning Score.

## Conclusion

Despite national recognition of the need for pregnant women to be assessed and treated by acute physicians with training in obstetric medicine during an acute medical illness, there is considerable variability in services available to patients from hospital to hospital and in availability of local clinical guidelines. The development of regional maternal medicine networks should ensure each region has access to appropriate training and clinical advice, improving care for this cohort of patients at each centre.
